# Supplementary material for: Structure of Venezuelan equine encephalitis virus in complex with the LDLRAD3 receptor
Source: Nature. 2021 Oct 13;598(7882):672–6. doi: 10.1038/s41586-021-03963-9 (PMC8550936; doi:10.1038/s41586-021-03963-9)
Supplement: Supplementary file 1 — Supplementary Tables 1–4 [file 41586_2021_3963_MOESM1_ESM.pdf]

nature portfolio

https://doi.org/10.1038/s41586-021-03963-9

Supplementary information

Structure of Venezuelan equine encephalitis virus in complex with the LDLRAD3 receptor

In the format provided by the authors and unedited

Supplementary Table S1. Summary of Cryo-EM data collection.

Sample	VLP alone[a] (EMDB 24117)	VLP + LDLRAD3 D1[a] (EMDB 24116)	VLP + LDLRAD3 D1D2[b] (EMDB 24394)
**# of Micrographs**	2237	8979	6693
**# of particles picked**	19516	30242	47820
**# of particles after 2D classification**	9816	14648	19115
**# of particles after 3D classification**	7993	12216	9634
**Resolution$_{FSC=0.5}$ (Å)**	4.75	5.03	6.49
**Resolution$_{FCS=0.143}$ (Å)**	4.23	4.28	4.97

[a]The microscope settings for image collection were: Dose: 35 $e^-/Å^2$; Magnification: 105,000x; Pixel size: 1.1; Voltage: 300 keV. Movies were recorded using a K2 Summit electron detector (Gatan).
[b]The microscope settings for image collection were: Dose: 35 $e^-/Å^2$; Magnification: 59,000x; Pixel size: 1.16; Voltage: 300 keV. Movies were recorded using a Falcon 4 Direct Electron Detector.

Supplementary Table S2. Refinement and model statistics.

**Asymmetric Unit Model**		**VLP alone (PDB 7N1I)**	**VLP + LDLRAD3(D1) (PDB 7N1H)**
**# of chains**		12	16
**# of residues**		4108	4264
**# of carbohydrates**		8	8
**# of CA ions**		0	4
**Resolution (Å)**		4.2	4.3
**MolProbity score**		1.87	1.90
**All-atom clash score**		8.33	8.79
**Rotamer outliers (%)**		0.00	0.00
**$C\beta$ outliers (%)**		0.00	0.00
**Ramachandran Plot values**	Favored (%)	93.63	93.48
	Allowed (%)	5.38	5.77
	Outliers (%)	0.78	0.76
**R.M.S. Deviations**	Bond lengths (Å)	0.002	0.002
	Bond angles (°)	0.573	0.572

Supplementary Table S3. List of contact residues at the VEEV E2-E1 and LDLRAD3(D1) binding interface

E2-E1 heterodimer	E2-E1 domain	E2-E1 residues	LDLRAD3(D1) residues
wrapped	Domain A of E2	V24, G25, S26, C27, H28, M70, H71, K116, S118, V119	C29, N34, N39, R41, C42, I43, P44, W47, L52, D54, F56
	Domain B of E2	S176, S177, K223	S38, D57, K62
	Fusion loop of E1	Y85, F87, M88, W89, G90, G91, A92, K225	M36, S38, N39, G40, R41, C55, F56, D57
intraspike	Domain A of E2	L5, G63, R64, L79, I92, V93, D94, G95	C42, I43, P44, G45, A46, W47, D50, G51, L52
	β-linker of E2	E148, V153, Y154, A155, H156, D157, A158, Q159, A262, D263, G264, K265, C266, T267	E28, C29, N30, I31, P32, G33, N34

Contact residues were identified using PDBePISA (www.ebi.ac.uk/pdbe/pisa/).

Supplementary Table S4. LDLRAD3 substitutions determined by BLOSUM scoring matrix

Position	Original Residue:	Mutate to:
**18**	Q	K
**19**	L	D
**20**	L	S
**21**	P	Y
**22**	G	D
**23**	N	K
**24**	N	E
**25**	F	D
**26**	T	E
**27**	N	L
**28**	E	R
**29**	C	
**30**	N	E
**31**	I	R
**32**	P	D
**33**	G	D
**34**	N	Y
**35**	F	K
**36**	M	T
**37**	C	
**38**	S	K
**39**	N	T
**40**	G	K
**41**	R	E
**42**	C	
**43**	I	
**44**	P	R
**45**	G	E
**46**	A	K
**47**	W	
**48**	Q	V
**49**	C	
**50**	D	V
**51**	G	H
**52**	L	D
**53**	P	D
**54**	D	
**55**	C	
**56**	F	D
**57**	D	V
**58**	K	D
**59**	S	R
**60**	D	
**61**	E	
**62**	K	E
**63**	E	K
**64**	C	
**65**	P	D

**66**	K	Y
**67**	A	R
**68**	K	E
**69**	S	H
**70**	K	E

The amino acids important for the structural integrity of LDLRAD3 protein (the disulfide bond-forming cysteines, residues coordinating calcium, or those buried in the hydrophobic core) were not mutated and are indicated in red.
