# Supplementary material for: Structure of Venezuelan equine encephalitis virus in complex with the LDLRAD3 receptor
Source: Nature. 2021 Oct 13;598(7882):672–6. doi: 10.1038/s41586-021-03963-9 (PMC8550936; doi:10.1038/s41586-021-03963-9)
Supplement: Supplementary file 2 — Reporting Summary [file 41586_2021_3963_MOESM2_ESM.pdf]

nature research

Corresponding author(s): Daved Fremont

Last updated by author(s): July 7, 2021

Reporting Summary

Nature Research wishes to improve the reproducibility of the work that we publish. This form provides structure for consistency and transparency in reporting. For further information on Nature Research policies, see our Editorial Policies and the Editorial Policy Checklist.

Statistics

For all statistical analyses, confirm that the following items are present in the figure legend, table legend, main text, or Methods section.

n/a	Confirmed	
☐	☒	The exact sample size (*n*) for each experimental group/condition, given as a discrete number and unit of measurement
☐	☒	A statement on whether measurements were taken from distinct samples or whether the same sample was measured repeatedly
☐	☒	The statistical test(s) used AND whether they are one- or two-sided<br>*Only common tests should be described solely by name; describe more complex techniques in the Methods section.*
☒	☐	A description of all covariates tested
☒	☐	A description of any assumptions or corrections, such as tests of normality and adjustment for multiple comparisons
☐	☒	A full description of the statistical parameters including central tendency (e.g. means) or other basic estimates (e.g. regression coefficient) AND variation (e.g. standard deviation) or associated estimates of uncertainty (e.g. confidence intervals)
☐	☒	For null hypothesis testing, the test statistic (e.g. *F*, *t*, *r*) with confidence intervals, effect sizes, degrees of freedom and *P* value noted<br>*Give P values as exact values whenever suitable.*
☒	☐	For Bayesian analysis, information on the choice of priors and Markov chain Monte Carlo settings
☒	☐	For hierarchical and complex designs, identification of the appropriate level for tests and full reporting of outcomes
☒	☐	Estimates of effect sizes (e.g. Cohen's *d*, Pearson's *r*), indicating how they were calculated

Our web collection on statistics for biologists contains articles on many of the points above.

Software and code

Policy information about availability of computer code

Data collection	FEI vitrobot (Thermo Fisher), Titan Krios (FEI), K2 summit electron detector (Gatan), Falcon 4 Direct Electron Detector (Thermo Fisher), EPU (Thermo FIsher), Biacore T200 system (GE Healthcare), TriStar Microplate Reader (Berthold Technologies), MACSQuant Analyzer 10 (Miltenyi Biotec).
Data analysis	MotionCor2 v1.4, Gctf v1.06, crYOLO v1.7.6, RELION v3.1, DeepEMhancer v20200909 , ChimeraX v1.3 , SWISS-MODEL (https://swissmodel.expasy.org), COOT v0.9.5, PHENIX v1.19.2-4158, PDBePISA (https://www.ebi.ac.uk/pdbe/pisa), FlowJo (BD, v10.7.0), BIAevaluation v3.1 (GE Healthcare). Prism (GraphPad, v8.4.3), PROMALS3D (https://prodata.swmed.edu/promals3d), ESPript v3.0.

For manuscripts utilizing custom algorithms or software that are central to the research but not yet described in published literature, software must be made available to editors and reviewers. We strongly encourage code deposition in a community repository (e.g. GitHub). See the Nature Research guidelines for submitting code & software for further information.

Data

Policy information about availability of data

All manuscripts must include a data availability statement. This statement should provide the following information, where applicable:

- Accession codes, unique identifiers, or web links for publicly available datasets
- A list of figures that have associated raw data
- A description of any restrictions on data availability

The authors declare that all data supporting the findings of this study are available within the paper and its Supplementary information and are available from the corresponding author upon request. All structures are deposited in the PDB and EMDB databases (PDB 7N1I, 7N1H; EMDB 24117, 24116, 24394). GenBank sequences used in the structural alignment analysis: VEEV strain TC-83, AAB02517; VEEV strain TrD, AAC19322; VEEV strain INH9813, AJP13627; VEEV strain ZPC738, AUV65225, EEEV strain FL93-939, ABL84687; WEEV strain CBA87, ABD98014; SINV strain Girdwood, AUV65223; CHIKV strain 37997, ABX40011.

Field-specific reporting

Please select the one below that is the best fit for your research. If you are not sure, read the appropriate sections before making your selection.

☒ Life sciences ☐ Behavioural & social sciences ☐ Ecological, evolutionary & environmental sciences

For a reference copy of the document with all sections, see nature.com/documents/nr-reporting-summary-flat.pdf

Life sciences study design

All studies must disclose on these points even when the disclosure is negative.

Sample size	No sample sizes were chosen a priori. All experiments were repeated at least three independent times, each with multiple technical replicates.
Data exclusions	No data was excluded.
Replication	All experiments had at least 3 independent biological replicates. All replication attempts were successful.
Randomization	No randomization was necessary as no human or animal subjects were used in the study.
Blinding	No blinding was necessary as no human or animal subjects were used inn the study.

Reporting for specific materials, systems and methods

We require information from authors about some types of materials, experimental systems and methods used in many studies. Here, indicate whether each material, system or method listed is relevant to your study. If you are not sure if a list item applies to your research, read the appropriate section before selecting a response.

Materials & experimental systems

n/a	Involved in the study
☐	☒ Antibodies
☐	☒ Eukaryotic cell lines
☒	☐ Palaeontology and archaeology
☒	☐ Animals and other organisms
☒	☐ Human research participants
☒	☐ Clinical data
☒	☐ Dual use research of concern

Methods

n/a	Involved in the study
☒	☐ ChIP-seq
☐	☒ Flow cytometry
☒	☐ MRI-based neuroimaging

Antibodies

Antibodies used	Anti-VEEV mAbs: 1A4A1-1, 3B4C-4, TRD-14, 57; Rabbit anti-FLAG antibody (Cell Signaling Technology, clone D6W5B, Cat #14793S); horseradish peroxidase (HRP)-conjugated goat anti-human IgG (H+L; Jackson ImmunoResearch, Cat #109-035-003).
Validation	Antibodies were validated by SDS-PAGE analysis and binding to viral recombinant proteins and/or infected cells. Many of these antibodies were sequence confirmed, generated in our laboratories, and previously used for similar applications (PMID: 33208938).<br>1. 1A4A-1 (Validated by SDS-PAGE analysis and binding to VEEV-infected cells and VEEV recombinant proteins); PMID: 2414905<br>2. 3B4C-4 (Validated by SDS-PAGE analysis and binding to VEEV-infected cells and VEEV recombinant proteins); PMID: 2414905<br>3. TRD-14 (Validated by SDS-PAGE analysis and binding to VEEV-infected cells and VEEV recombinant proteins); N.M.K. and M.S.D., unpublished<br>4. 57 (Validated by SDS-PAGE analysis and binding to VEEV-infected cells and VEEV recombinant proteins); N.M.K. and M.S.D., unpublished<br>5. Rabbit anti-FLAG (Cell Signaling Technology, clone D6W6B, Cat #14793S); Commercially validated by flow cytometry<br>6. Horseradish peroxidase (HRP)-conjugated goat anti-human IgG (H+L; Jackson ImmunoResearch, Cat #109-035-003); Commercially validated by ELISA

Eukaryotic cell lines

Policy information about cell lines

Cell line source(s)	Neuro-2a (Cat #CCL-131) cells were obtained from ATCC. Expi293F (Cat #A14527) cells were obtained from Thermo Fisher.
Authentication	These cells were obtained from ATCC or other commercial vendors and grew and performed as expected. Morphology of each cell line was assessed by microscopy.

Mycoplasma contamination	All cell lines are routinely tested each month and were negative for mycoplasma.
Commonly misidentified lines (See ICLAC register)	This study did not involve any commonly misidentified cell lines.

Flow Cytometry

Plots

Confirm that:

☒ The axis labels state the marker and fluorochrome used (e.g. CD4-FITC).

☒ The axis scales are clearly visible. Include numbers along axes only for bottom left plot of group (a 'group' is an analysis of identical markers).

☒ All plots are contour plots with outliers or pseudocolor plots.

☒ A numerical value for number of cells or percentage (with statistics) is provided.

Methodology

Sample preparation	After infection, cells were harvested, fixed, permeabilized (or non-permeabilized in some experiments), and stained with the antiviral or anti-FLAG antibodies described above.
Instrument	MACSQuant Analyzer 10
Software	FlowJo software (BD)
Cell population abundance	rans-complemented cells were analyzed for transgene expression using anti-FLAG antibody
Gating strategy	Gating was performed based on non-binding control antibodies and/or uninfected cells. Dead cells were excluded by scatter and size.

☒ Tick this box to confirm that a figure exemplifying the gating strategy is provided in the Supplementary Information.
